# Egrets Promote the Transmission and Spread of Plasmid‐Mediated Colistin Resistance Gene *mcr-1*‐Bearing *Escherichia coli* Strains in Crested Ibis

**DOI:** 10.1155/tbed/4384955

**Published:** 2026-06-07

**Authors:** Keyuan Chen, Wulin Ma, Lei Lei, Guoqiang Qiu, Jian Shi, Ying Li, Yadong Zheng, Weihuan Fang, Houhui Song, Yongchun Yang

**Affiliations:** ^1^ Key Laboratory of Applied Technology on Green-Eco-Healthy Animal Husbandry of Zhejiang Province, College of Veterinary Medicine, Zhejiang A&F University, Hangzhou, Zhejiang, China, zafu.edu.cn; ^2^ Deqing County Bureau of Natural Resources and Planning, Deqing, 313200, Zhejiang Province, China; ^3^ Huzhou Ecological Forestry Protection Research Center, Huzhou, 313000, Zhejiang Province, China; ^4^ College of Animal Sciences, Zhejiang University, Hangzhou, 310058, Zhejiang Province, China, zju.edu.cn

**Keywords:** antimicrobial resistance gene, crested ibis, egret, spread

## Abstract

Colistin resistance genes have attracted increasing global attention due to their plasmid‐mediated transmissibility. To clarify the transmission dynamics of colistin resistance genes in the endangered crested ibis (*Nipponia nippon*) and its environment, surveillance for *mcr-1* was conducted at a captive breeding center in Deqing, Zhejiang, China, from 2017 to 2024. Genomic sequencing was used to analyze the phylogenetic relationships among the Enterobacteriaceae isolates harboring these resistance genes. A total of 2660 fecal samples of crested ibises were collected seasonally. There was only one episode of *mcr-1* occurrence with a total of 144 *mcr-1*‐positive strains in crested ibises. The prevalence was first detected at 8% in April 2021, surged to 25% in July, and then declined to 10.6% in October. Additionally, *mcr-1*‐harboring *Escherichia coli* isolates were also found in feces from wild egrets (33%), the staff member’s dog (8.3%), and effluent sewage, but not in the staff member, loach samples, or water sources. Phylogenetic analysis showed that the egret‐derived isolate BLM86 and the crested ibis‐derived isolate ZHM12 belonged to the same ST58‐cgST28300 type; no core‐genome SNP differences were detected. All 36‐deeper analyzed *mcr-1*‐positive isolates displayed multidrug resistance, with four distinct *mcr-1*‐containing plasmid types identified: IncI2 (20/36, 55.6%), IncX4 (19.4%), IncP1 (11.1%), and IncHI2 (13.9%). Phylogenetic analysis indicated that several of these crested ibis‐derived isolates were closely related to those from egrets, implying potential environmental transmission routes to both the crested ibis and egrets. *Mcr-1* was no longer detected in the crested ibis population after implementation of biosecurity interventions. Our findings demonstrate that wild egrets could act as potential vectors for the environmental transmission of *mcr-1* to captive crested ibises, highlighting the importance of interspecies barriers in antimicrobial resistance (AMR) containment.

## 1. Introduction

Colistin, an antimicrobial of the polymyxin class, is considered a last‐line treatment for multidrug‐resistant (MDR) infections, particularly those caused by carbapenemase‐producing Gram‐negative bacteria [1]. However, its effectiveness is increasingly threatened by the rise of colistin resistance, posing a growing global health risk [2]. Due to its toxicity and poor renal clearance, the use of colistin in both humans and food animals is restricted [3]. Of particular concern is the spread of resistance in wildlife, including wild birds, which may be a significant yet underappreciated vector in the transmission of antimicrobial‐resistant bacteria (ARB) [4]. This also represents a potential contamination risk for human and animal habitats. Consequently, a comprehensive understanding of the mechanisms underlying colistin resistance, along with strategies to curb its spread, is critical.

The plasmid‐mediated *mcr-1* gene, responsible for colistin resistance, was first identified in China and has since been detected globally in various *Enterobacteriaceae* species in humans, animals, and environmental samples [5, 6]. Notably, animal‐derived isolates have shown a higher prevalence of *mcr-1* than those from human clinical settings, highlighting the role of animals as both sources and reservoirs of resistance [5]. Moreover, plasmids harboring the *mcr-1* gene exhibit diverse incompatibility genotypes (sizes: 32–400 kb), with IncI2 and IncX4 being the most prevalent [2, 7]. Additionally, the horizontal transfer of *mcr-1* is efficient, with minimal fitness costs, which further facilitates its cross‐species transmission [8].

The crested ibis (*Nipponia nippon*), one of the rarest avian species in the world, is classified as “Endangered” on the International Union for Conservation of Nature (IUCN) Red List [9]. Antimicrobial resistance (AMR) surveillance in endangered, captive populations like the crested ibis is particularly crucial. The introduction of MDR bacteria could compromise individual welfare, undermine conservation breeding efforts, and potentially translocate resistance into protected habitats upon reintroduction [10]. In April 2021, we detected *mcr-1*‐positive *Escherichia coli* in crested ibis fecal samples, indicating that colistin resistance could pose both ecological and public health risks [10]. Wild birds, as an integral part of the ecosystem, are widely distributed across diverse environments. Increasing evidence suggests that they can acquire ARB from contaminated habitats and disperse these pathogens along their migratory routes [11]. In particular, wild egrets usually undergo extensive seasonal migration in winter and summer, which has been associated with ecological issues such as heavy metal pollution [12]. Although *mcr-1* has been detected in wild birds worldwide, the mechanisms driving its transmission among birds, humans, and other animals remain inadequately understood [13, 14]. This knowledge gap highlights the need for a thorough investigation of the *mcr-1* gene spread within crested ibises’ populations to safeguard the ecological balance of their habitat.

The transmission pathways of *mcr-1* among wild birds, humans, and cohabiting animals remain still poorly resolved. The crested ibis breeding center in Deqing represents a unique environment with abundant wild egrets and limited human activity and a history of successful captive breeding. In this study, we hypothesized that wild egrets could act as mobile vectors, introducing *mcr-1* from external sources into this semiclosed system. To test this, we conducted the surveillance study to characterize the epidemiology of *mcr-1* in crested ibises, used high‐resolution genomics to trace potential transmission links between hosts, and assessed the impact of targeted interventions. Our findings highlight key risk factors for the acquisition of *mcr-1* by the intestinal bacteria of crested ibises and suggest the potential for broader dissemination of this resistance, offering valuable insights into the prevention and control of *mcr-1* transmission.

## 2. Methods

### 2.1. Sample Collection and Bacterial Isolation

The largest captive breeding base for crested ibises in China is located in Deqing County, Zhejiang Province, China, where the crested ibis population is the third largest in the country. From 2017 to 2024, a total of 2660 individual fecal samples from crested ibises were collected seasonally (spring, summer, autumn, and winter) for surveillance of the colistin resistance gene *mcr-1* in this population. The details of sample collection are as follows: (1) Individual ibises: fecal samples from crested ibis were collected seasonally (spring, summer, autumn, and winter). Each sample collected within 30 min after cleaning was considered to originate from an individual crested ibis. (2) Human: each staff member voluntarily provided one single fecal sample using a sterile container. (3) Dog: a single fecal sample from the staff member’s dog was collected from the ground using a sterile swab. (4) Wild egrets: fecal samples were collected from the ground between crested ibis enclosures at the breeding center and the egret habitats near the breeding facility. Sampling points were spaced more than 10 m apart to minimize the risk of resampling the same individual. (5) Water: 500 mL of drinking water was collected from the Ibis enclosure troughs into sterile bottles. (6) Sewage: 200 mL of effluent water was collected from the drainage outlet of the enclosure complex. (7) Loach: intestinal contents were collected aseptically (Supporting Information 1: Table S1).

With the finding of *mcr-1*‐positive strains in the crested ibises, we conducted a cross‐sectional study from July to October 2021 to analyze the origin of the *mcr-1* gene in the crested ibis. A total of 143 samples were collected, including wild egret feces (*n* = 21 in July), staff feces (*n* = 9 in October), domestic dog feces (*n* = 12 in July), the primary feed (loach, *n* = 60 per sampling in July and October), drinking water (*n* = 5 per sampling), and effluent sewage (*n* = 15 in July and *n* = 21 in October). To inhibit the spread of *mcr-1*, the biosafety intervention strategies adopted in this study include enhanced daily disinfection of enclosures and facilities using chlorine‐based disinfectants; installation of bird deterrent nets around the breeding cages to reduce wild bird (especially egret) intrusions; strict hygiene protocols for staff entering/exiting enclosures, including footwear disinfection and use of dedicated clothing; and removal of accumulated egret droppings from the vicinity of the crested ibises enclosures.

All samples were cultured in EE (Enterobacteriaceae enrichment) broth (Thermo Scientific, CM0317B) containing 4 mg/L colistin for 6 h, followed by polymerase chain reaction (PCR) to identify the presence of *mcr-1* [5]. Positive samples were then cultured on China blue agar plates (Hopebiol, HBPM6233) containing 4 mg/L colistin for 12 h [15]. Randomly, 10 or more different colonies were selected from each plate and incubate them in Luria Bertani broth overnight at 37°C and 200 rpm. Further identification was performed using PCR and sequencing of the 16S rRNA gene. The bacterial solution will then be mixed with 50% glycerol and frozen at 80°C. This methodological choice may have led to an underestimation of the true prevalence of *mcr-1*‐positive isolates, particularly those exhibiting low‐level resistance (e.g., MIC = 2 mg/L) [16].

Throughout the period of surveillance, no polymyxins were used in any of the populations surveilled, including the crested ibises, breeders, loaches, drinking water, livestock, or poultry. After discovering the presence of *mcr-1* in the crested ibis population, we implemented control measures, including enhanced environmental disinfection, removal of wild birds around the breeding cages, and regulations governing the behavior of employees entering and leaving the cages, in order to limit the spread of *mcr-1*.

### 2.2. Whole Genome Analysis

To assess the genomic and epidemiological relationships among the *mcr-1* positive isolates, 36 representative strains were selected for whole‐genome sequencing (WGS). Total genomic DNA was extracted from each strain using the DNeasy PowerSoil Pro Kit (QIAGEN). Libraries were constructed with the NEXT Ultra DNA Library Preparation Kit (QIAGEN), and 400 bp paired‐end reads were generated using the Illumina NovaSeq 6000 platform (Illumina, San Diego, CA, USA). For each isolate, at least 100‐fold coverage was achieved. Genomic assemblies were performed using SPAdes Version 3.9.0 [17]. Phylotyping of the *Escherichia* genus strain was conducted using the ClermonTyping method [18].

Antimicrobial resistance genes (AMRGs), multilocus sequence typing (MLST), and core‐genome multilocus sequence typing (cgMLST) were analyzed using the Center for Genomic Epidemiology platform (https://cge.cbs.dtu.dk//services/) with the assembled genomes [19, 20]. Virulence factors (VFs) were annotated using the Virulence Factor Database (VFDB) [21]. Plasmid types carrying the *mcr-1* gene (Inc types) were determined using PlasmidFinder 2.1 [22]. For contigs with ambiguous plasmid typing results, BLASTN was used for sequence homology search to obtain high‐identity sequences, followed by confirmation with PlasmidFinder 2.1. The genetic backgrounds of different *mcr-1 carrying* plasmids were annotated using Prokka (https://proksee.ca/).

### 2.3. Construction of a Core Genome‐Based Phylogenetic Tree

The phylogenetic tree‐based core‐genome single nucleotide polymorphisms (SNPs) of 36 *mcr-1*‐positive Enterobacteriaceae isolates were constructed using components from Harvest Version 1.1.2 [23]. Specifically, the phylogenetic tree was constructed using the maximum likelihood method implemented in Harvest v1.1.2, with 1000 bootstrap replicates.

To expand the comparative scope of the phylogenetic analysis, an additional 99 *mcr-1*‐positive *Escherichia coli* were retrieved from NCBI for inclusion in this study. The selection criteria were as follows: (i) the strains were associated with waterfowl or aquatic environments and (ii) complete background information was available for all selected strains. These selected strains comprised 60 avian strains (13 from chickens and 47 from ducks), 21 human strains, and 18 strains from other sources (1 dog, 2 fish, 8 environmental, 4 flies, and 3 pigs). Phylogenetic tree lineages were defined using Rhierbaps, and the source of each isolate was visualized using iTOL (https://itol.embl.de) [24–26].

### 2.4. Antimicrobial Susceptibility Testing

The colistin minimal inhibitory concentration (MIC) for each isolate was determined using the broth microdilution method as recommended by the European Committee on Antimicrobial Susceptibility Testing (EUCAST, Clinical Breakpoint Tables v. 12.0). The susceptibility of *mcr-1*‐positive isolates to a panel of antimicrobial agents was further assessed using the Kirby‐Bauer disc diffusion method (Supporting Information 2: Table S2), and the results were interpreted according to the Clinical and Laboratory Standards Institute (CLSI M100‐Ed31) criteria. The *E. coli* strain ATCC25922 served as the control.

### 2.5. Data Analysis

Statistical comparisons of *mcr-1* prevalence were performed between (i) different sampling months within the crested ibis population; (ii) crested ibis and egrets within the same sampling period (July 2021); and (iii) all host types (crested ibis, egret, human, and dog) present in the July–October 2021 cross‐sectional surveillance. All statistical analyses were performed using IBM SPSS Statistics 20. Differences in the *mcr-1* carrier rate were evaluated using the *χ* ^2^ test (for *n* ≥ 50) or Fisher’s exact test (for *n* < 50). The *p*‐values were adjusted for multiple comparisons using the Bonferroni method. A *p*‐value of less than 0.05 was considered statistically significant.

## 3. Results

### 3.1. Prevalence of Colistin Resistance Gene *mcr-1* in Crested Ibis

**Figure 1:**
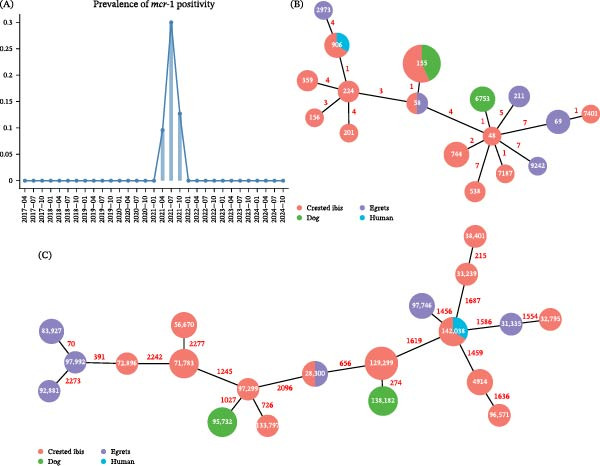
Prevalence and molecular characterization of *mcr*‐*1*‐positive isolates. (A) prevalence of *mcr-1* in crested ibises during the surveillance period (2017–2024). (B) Minimum spanning tree (MST) of *mcr-1*‐positive isolates based on multilocus sequence typing (MLST) data. (C) Minimum spanning tree (MST) of *mcr-1*‐positive isolates based on core‐genome multilocus sequence typing (cgMLST) data. In B and C, different colors represent the isolates sources, and node sizes are proportional to the number of isolates per sequence type (ST).

To assess the prevalence of the colistin resistance gene *mcr-1* in crested ibises, a total of 2,660 fecal samples were collected from crested ibises in Deqing County between 2017 and July 2024 (Supporting Information 1: Table S1). We found that *mcr-1* was first detected in April 2021, with a positive rate of 8% (8/100, 95% CI: 3.5%–15.2%). The detection rate surged to 25% (17/68, 95% CI: 15.3%–37.0%, *p* = 0.04) in July and then dropped to 10.6% (9/85, 95% CI: 4.9%–19.2%, *p* = 0.29) in October. Notably, no *mcr-1*‐positive samples were found in crested ibises from January to July 2022 (Figure 1A; Supporting Information 3: Table S3).

Following the initial detection of *mcr-1* in the crested ibis feces, a cross‐sectional survey was conducted between April and July of 2021. Samples were collected from the food, drinking water, and sewage associated with the crested ibises, as well as from the feces of egrets, keepers, and their domestic animals. Fecal samples from egrets, collected in July, showed an *mcr-1*‐positive rate of 33% (7/21, 95% CI: 14.6% – 57.0%, Supporting Information 3: Table S3). The *mcr-1* was also detected in the feces of a staff member’s dog, with a positive rate of 8.3% (1/12, 95% CI: 0.21–38.5, Supporting Information 3: Table S3), but was not detected in the feces of the worker, the intestinal samples of loach, or the drinking water (Supporting Information 3: Table S3). These findings suggest that the *mcr-1*‐positive strains in the crested ibises might not be acquired through food or drinking water. Additionally, *mcr-1* was detected in the effluent sewage, with a positive rate of 40% (6/15, 95% CI: 16.3%–67.7%) in July and 52.4% (11/21, 95% CI: 29.8%–74.3%) in October (Supporting Information 3: Table S3). Notably, during follow‐up surveillance in July 2022, *mcr-1* was again detected in fecal samples from egrets and from a family of a previous staff member, but no positive samples were found in crested ibises (Supporting Information 3: Table S3). Taken together, these results suggest that the wild egret may be an important vector for the transmission of *mcr-1*.

### 3.2. Genomic Epidemiology of *mcr-1*‐Positive Isolates

**Figure 2:**
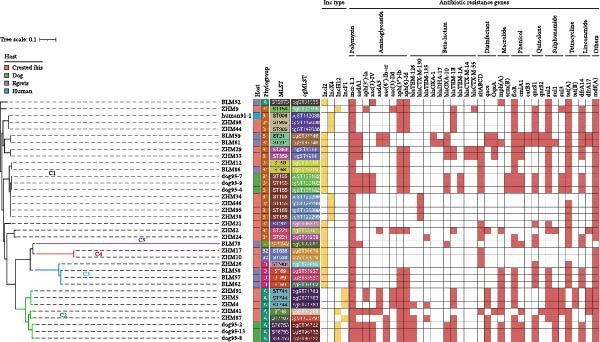
Phylogenetic tree of the 36 *mcr-1*‐positive *Escherichia coli* and *Escherichia fergusonii* isolates and their association with other antimicrobial genes. The phylogenetic tree was constructed based on core‐genome single nucleotide polymorphism (SNP) data and was rooted using the midpoint method. Branch colors represent distinct genetic lineages, labeled as C1–C5. For each isolate, the following information is provided after the isolate name: host, phylogroup, multilocus sequence type (MLST), and core‐genome multilocus sequence typing (cgMLST) type. The molecular characteristics of each isolate, including Inc type and resistance genes, are represented by squares: empty squares indicate the absence of a feature, while filled squares indicate the presence of a feature. Each column corresponding to resistance genes is annotated with the specific genes and their associated functions.

A total of 144 *mcr-1*‐positive isolates were obtained from the collected samples (Supporting Information 4: Table S4). All isolates were identified as *E. coli*, except for one strain (BLM78), which was identified as *Escherichia fergusonii* from an egret sample. To assess the genetic relationships among the *mcr-1*‐positive isolates, 36 isolates were selected from different hosts for WGS analysis: crested ibis (*n* = 21), human (*n* = 1), dog (*n* = 6), and egret (*n* = 8). The MLST analysis based on WGS data revealed that the 36 *mcr-1*‐positive isolates comprised 17 different sequence types (STs). The most common types were ST155 (*n* = 7), ST69 (*n* = 3), ST744 (*n* = 3), ST906 (*n* = 3), and ST6753 (*n* = 3) (Figure 1B and Figure 2). Within these lineages, we observed both host‐specific and cross‐host genotypes. For example, the ST155 strain coexisted in both crested ibises and dogs, the ST906 strain was found in both crested ibis and human isolates, and the ST58 strain was shared between egret and crested ibis samples (Figure 1B). The cgMLST analysis classified the 36 isolates into 20 cgST types, with 1–4 strains per type. The number of allelic differences between strains ranged from 0 to 2273, with cgST129299 (*n* = 4) being the dominant type (Figure 1C and Figure 2). Mostly, strains within the same cgMLST type shared the same ST type. However, ST155 comprised strains from both cgST129299 and cgST138182 (Figure 1C).

To further explore the genetic relationships among the isolates, a phylogenetic tree was constructed using core genomic SNPs. Bayesian analysis of population structure (BAPS) defined five distinct lineages (C1–C5). Lineage C1 was the most common, comprising 21 isolates (58.3%, 21/36) from crested ibis, egret, human, and dog samples (Figure 2). Notably, no core‐genome SNPs were detected between BLM86 and ZHM12, which belong to the same ST58‐cgST28300 type (Figure 2). In addition, ClermonTyping of the isolates classified them into five phylogroups: phylogroup A (*n* = 9), phylogroup B1 (*n* = 20), phylogroup B2 (*n* = 2), phylogroup D (*n* = 4), and one isolate as *E. fergusonii* (*n* = 1, shown as “‐”) (Figure 2). Phylogroup B1 was the predominant group and was detected across all sources in this study. Except for the egret isolate BLM52, the results from ClermonTyping were consistent with those obtained from the Bayesian analysis (Figure 2).

The molecular diversity observed in this study, along with the potential for cross‐species transmission, suggests that the *mcr-1* colistin resistance gene may spread between animals and humans through various environmental carriers associated with the crested ibis. In particular, wild egrets play an important role in the transmission of *mcr-1*.

### 3.3. AMR Analysis of *mcr-1*‐Positive Isolates

Susceptibility testing of 36 *mcr-1*‐positive isolates was conducted using 14 antimicrobials. The results revealed that all isolates were resistant to more than four antimicrobials, with 100% resistance observed for neomycin, cefradine, polymyxin B, and tetracycline (Table 1). Furthermore, after analyzing the association between drug resistance phenotypes and antibiotic resistance genes, we found that the universal resistance to tetracycline correlated with the high prevalence of the *tet*(*A*) efflux pump gene. Similarly, resistance to β‐lactams like ampicillin was associated with genes such as *bla*TEM‐1B and *bla*CTX‐M variants. The presence of *qnrS1* and aminoglycoside‐modifying enzyme genes (*aph* and *aac*) aligned with the resistance profiles for quinolones and aminoglycosides, respectively. These findings demonstrate that *mcr-1*‐positive isolates are MDR.

**Table 1:** Drug sensitivity results of the *mcr-1*‐positive isolates.

Classification	Antibiotics	Resistance isolates (%, 95%CI)
Aminoglycosides	Gentamicin	12 (33.3, 18.6–51.0)
	Kanamycin	18 (50, 32.9–67.1)
	Neomycin	36 (100)
Beta‐lactam	Cefepime	14 (38.9, 23.1–56.5)
	Cefixime	13 (36.1, 20.8–53.8)
	Cefradine	36 (100)
	Ampicillin	29 (80.6, 70.5–95.3)
	Carbenicillin	30 (83.3, 67.2–93.6)
Quinolone	Levofloxacin	19 (52.8, 35.5–69.6)
	Ciprofloxacin	31 (86.1, 70.5–95.3)
Phenicol	Chloramphenicol	27 (75, 57.8–87.9)
	Florfenicol	27 (75, 57.8–87.9)
Polypeptide	Polymyxin B	36 (100)
Tetracyclines	Tetracycline	36 (100)

### 3.4. Genomic Characteristics of *mcr-1*‐Positive Isolates

To assess the carriage of ARGs and VFs in *mcr-1*‐positive isolates, a genetic library of ARGs (87 isoforms) and VFs (97 isoforms) was constructed based on WGS data. Resistance genes for tetracycline, rifamycin, quinolones, polymyxin, amphenicol, and aminoglycosides were detected in all isolates (Figure 2; Supporting Information 5: Figure S1A). Among these, Mdf (A), which encodes a multidrug efflux pump in *E. coli*, was the most prevalent, present in 97.2% (35/36) of the isolates. Resistance genes for *tetracycline* (*tet* (*A*)), *florfenicol* (fIoR), *aminoglycosides* (*aph* (*3^′^’*)‐*Ib*, *aph* (*6*)‐*Id*), *sulfonamides* (*sul1*, *sul2*), *quinolones* (*qnrS1*), and *trimethoprim* (*dfrA14*) were also detected in more than 50% of the isolates. Due to widespread use of *β-lactam* antibiotics in both humans and animals, 77.8% (28/36) of the isolates harbored at least one class of β‐lactam resistance genes, including *beta-lactamase* (*blaTEM*, *blaCTX-M*, *blaDHA*, *blaOXA*, and *blaIAP*). In contrast, all isolates were negative for carbapenemase‐producing genes.

The VFs were predicted using the VFDB database (Supporting Information 5: Figure S1B). Genes associated with functions such as adherence, effector delivery systems, and nutritional/metabolic processes were abundant in the genomes, potentially facilitating prolonged colonization of *E. coli* in the host. Furthermore, exotoxin secretion genes (cdtA, cdtB, cdtC, and usp) were found exclusively in isolates ZHM10 and ZHM17. A negative correlation was observed between the distribution of virulence genes and the number of ARGs, with a correlation coefficient of −0.371 (*p* < 0.01). This finding suggests that strains carrying a higher number of ARGs may exhibit lower virulence.

### 3.5. Genetic Background of the Plasmids Harboring *mcr-1*

The presence and genetic localization of *mcr-1* were determined using PlasmidFinder 2.1, BLASTN, and mapping analysis based on WGS data. The most common plasmids harboring *mcr-1* were IncI2 (20/36, 55.6%) and IncX4 (7/36, 19.4%), together accounting for 75% of the isolates (27/36, 95% CI: 57.8% ~ 87.9%). Additionally, IncP1 (4/36, 11.1%) and IncHI2 (5/36, 13.9%) were also found to harbor *mcr-1* (Figure 2).

**Figure 3:**
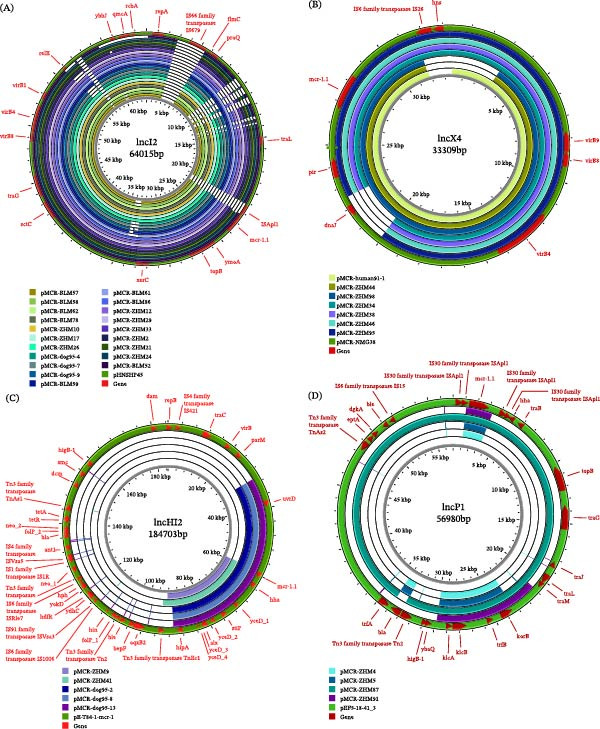
Comparison of reference plasmid sequences with homologous overlapping clusters from *mcr-1*‐positive isolates in this study. Each ring represents a corresponding plasmid, and the plasmid type is indicated at the center of each ring. (A) IncI2 plasmid (reference plasmid pHNSHP45, GenBank Accession Number KP347127); (B) IncX4 plasmid (reference plasmid pMCR‐NMG38, GenBank Accession Number MK836307.1); (C) IncP1 plasmid (reference plasmid pEF5‐18‐41_3, GenBank Accession Number CP063495.1); and (D) IncHI2 plasmid (reference plasmid pE‐T84‐1‐*mcr-1*, GenBank Accession Number CP090269.1).

The IncI2 plasmid was detected in isolates from crested ibis, egrets, and humans (Figures 2 and 3A). The flanking sequences of *mcr-1* in all 20 IncI2 plasmids showed >99% nucleotide identity and >95% query coverage to the corresponding region of the reference plasmid pHNSHP45 (IncI2; KP347127.1) when analyzed by BLASTN, which was the first plasmid identified to harbor *mcr-1* in pig‐derived *E. coli* from China [5]. Notably, the ISApI1 insertion sequence, which is located upstream of *mcr-1* in pHNSHP45, was absent in the isolates from this study (Figure 3A). Five plasmids (pMCR‐BLM57, pMCR‐BLM58, pMCR‐BLM59, pMCR‐BLM61, and pMCR‐BLM78) contained the ISSen6 insertion sequence, while pMCR‐BLM52 contained ISEc9, while other plasmids lacked any insertion sequences.

The IncX4 plasmid was found in isolates from both crested ibis and humans (Figures 2 and 3B). Seven IncX4‐related overlapping groups showed high homology, with their genetic background resembling that of plasmid pMCR‐NMG38 (IncX4; MK836307.1). Three of these plasmids (pMCR‐ZHM44, pMCR‐ZHM98, and pMCR‐human91) did not contain any insertion sequences. However, IS26 was identified ~3 kb upstream of *mcr-1* in the plasmids pMCR‐ZHM34, pMCR‐ZHM38, pMCR‐ZHM46, and pMCR‐ZHM95, although no other resistance genes were detected in any of the IncX4‐type plasmids.

The IncP1‐type plasmid was detected exclusively in crested ibis‐derived isolates, with a plasmid backbone similar to that of pEF5‐18‐41_3 (IncP1; CP063495.1) (Figure 3C). In plasmid pMCR‐ZHM87, ISApI1 elements were present both upstream and downstream of *mcr-1*. Finally, five IncHI2‐type plasmids were isolated from crested ibis and dog isolates, with no insertion sequences or additional resistance genes detected (Figure 3D). These data suggest that these four plasmids play an important role in the spread of *mcr-1* in this study, and wild egrets may transmit *mcr-1* to crested ibis through these four plasmids.

### 3.6. Associations Among the Isolates of Different Origins

**Figure 4:**
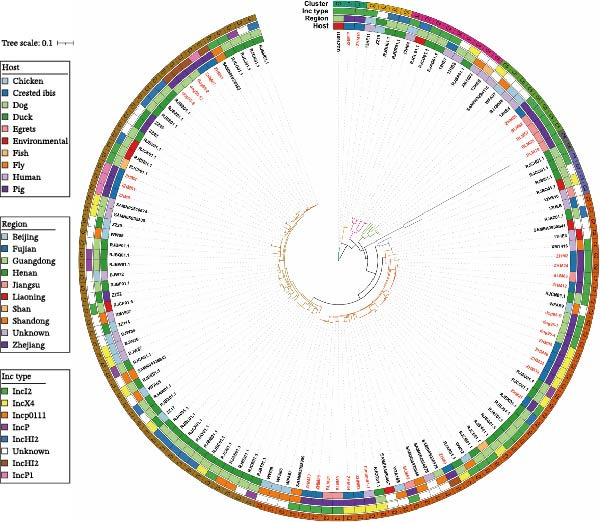
Phylogenetic tree of *mcr-1*‐positive *E. coli* from different sources. The midpoint tree was generated using core genomic SNP data. For each isolate, information on host, region, Inc type, and cluster is provided after the corresponding isolate names.

To further assess the potential transmission dynamics of *mcr-1*‐positive isolates, we downloaded whole‐genome sequences from 99 *mcr-1*‐positive isolates, which originated from diverse sources, including chickens, ducks, dogs, fish, and humans (Supporting Information 6: Table S5). A phylogenetic tree was constructed based on SNPs in the core genome, utilizing a total alignment size of ~679 Mb (~5 Mb per strain). The analysis revealed that isolates from the same source (host and region) typically clustered together, exhibiting similar genetic characteristics. However, the genetic context of the *mcr-1* gene varied across different Inc types (Figure 4; Supporting Information 6: Table S5). Among the 36 deeply analyzed isolates from this study, six distinct branches were formed within the broader phylogenetic tree, with 21 crested ibis‐derived isolates distributed across four branches (Figure 4). Notably, the majority (61.9%, 13/21) of crested ibis‐derived isolates were concentrated in the C2 branch, which also contained isolates from diverse hosts, including egrets, humans, and dogs (Figure 4). These results suggest that *mcr-1*‐positive *E. coli* strains have the potential to spread widely across both animal and environmental reservoirs. This transmission could play a significant role in colonization, dissemination through the food chain, and environmental exposure, contributing to a closed‐loop cycle of animal–environment–human transmission.

## 4. Discussion

**Figure 5:**
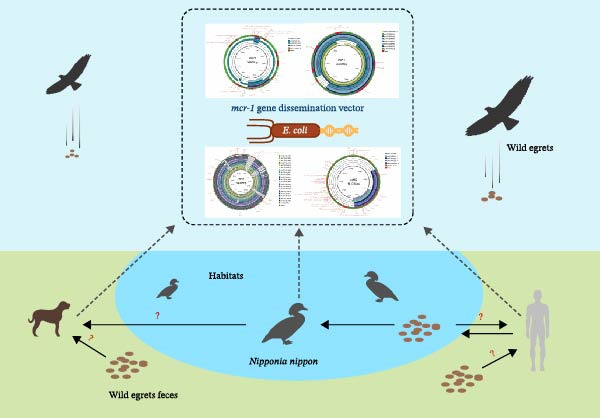
Proposed model for the environmental dissemination of the *mcr-1* gene based on study findings. The captive crested ibises acquire *mcr-1* from the habitat environment, while wild egrets pose a potential risk to the transmission of *mcr-1* during this process. The solid arrows indicate processes that are definitively occurring, while the dashed arrows represent processes that are potentially occurring.

The spread of mobile AMR genes poses a significant global threat to ecological balance as these genes are disseminated across widely dispersed microorganisms and their environments [6, 10]. In this report, we have found that a large number of *E. coli* isolates harboring the *mcr-1* resistance gene were present in the feces of the Crested ibis. WGS and cross‐sectional study further indicated that wild egrets may be a contributing vector in the transmission of *mcr-1* to crested ibises, dogs, and humans and that four plasmids: IncI2, IncX4, IncP1, and IncHI2, are involved in this transmission process (Figure 5).

Growing evidence suggests that wild birds can acquire ARB from contaminated environments and disseminate these bacteria along their migratory flight paths. For urban‐dwelling wild birds, which inhabit highly polluted urban settings, increased exposure to humans and contaminated environments enhances their likelihood of acquiring ARGs and becoming carriers of multiple ARG types [4]. We observed that the initial discovery of the plasmid‐mediated colistin resistance gene *mcr-1* in crested ibis feces in April 2021 was followed by a significant increase in its prevalence with its peak in July 2021, which then abruptly decreased. From January to July 2022, *mcr-1* was no longer detected in crested ibis feces samples, although it reappeared in feces samples from egrets and humans in July 2022. Following enhanced disinfection measures in the breeding environment and efforts to reduce egret droppings around the crested ibis enclosures in response to the initial finding of *mcr-1*, the prevalence of this particular ARG declined rapidly and was completely eliminated within 6 months after the egret control measures were implemented. This pattern coincides with the rapid decline in *mcr-1* prevalence following the prohibition of colistin use as an animal feed additive [27]. This suggests that reducing or even eliminating the carrier rate of *mcr-1* may help decrease colistin resistance in both humans and livestock. Such efforts are crucial for maintaining the ecological health of the crested ibis habitat.

During migration, wild birds traverse diverse geographical regions and interact with ARG‐harboring bacteria across a variety of environments and biotic communities, thereby acquiring a broad repertoire of ARGs through activities such as foraging. Additionally, wild birds often congregate in large flocks during migration, and this high‐density grouping also promotes the frequent transmission of ARGs [28]. In this study, we investigated the phylogeny and genome‐wide features of *E. coli* strains carrying the *mcr-1* gene. Our findings revealed that isolates from various host sources exhibited genetic diversity and multidrug resistance, along with a substantial library of ARGs and VFs. The 36 deeply analyzed isolates exhibited genetic diversity and multidrug resistance, with four phylogroups (A, B1, B2, and D) and *E. fergusonii* identified. The phylogroup B1, commonly found in vertebrates and generally nonpathogenic or symbiotic [29, 30], was the most prevalent. These isolates represented 17 STs and 20 cgSTs. Despite high clonality, the presence of identical MLST and cgMLST types across different host species suggested the circulation of closely related strains across hosts. Notably, ST155 (phylogroup B1) occurred in both dogs and crested ibises and has been reported in Chinese poultry and Portuguese deer [31, 32]. More specifically, isolates BLM86 (from an egret) and ZHM12 (from a crested ibis) were phylogenetically close (ST58‐cgST28300), indicating a strong genetic link [33]. Collectively, these results suggest a closed‐loop cycle of animal–environment–human transmission, in which *mcr-1*‐positive *E. coli* strains have the potential to spread widely across both animal and environmental reservoirs. Egrets, as urban‐dwelling waterbirds, frequently inhabit polluted rivers and surrounding areas, and the *mcr-1* gene has been detected in *E. coli* from both polluted river water and egret feces, suggesting that egrets mediate AMR transmission from polluted water to park environments [28, 34]. Since egrets and crested ibises inhabit the same habitat, with egret feces commonly found around ibis enclosures, and given the limited range of captive ibises with no direct contact with poultry or livestock, the most plausible route for crested ibises to acquire *mcr-1* is from egrets. Animal keepers may also act as *mcr-1* carriers by interacting with the surrounding environment. Therefore, these findings highlight the importance of tracing the origin and transmission pathways of resistance genes in wild birds. Although the direction of *mcr-1* transmission cannot be definitively determined with absolute certainty, multiple lines of evidence support that crested ibises most likely acquired the gene from egrets: *mcr-1* was consistently negative in crested ibises before 2021; a small outbreak occurred in 2021; water, loach, and other environmental samples from their habitat were all negative; and importantly, after implementing intervention measures that specifically targeted egrets (without altering the crested ibises’ rearing environment, water and food sources, or daily management), the *mcr-1* positivity in crested ibises gradually turned negative.

The *mcr-1* gene, first identified in IncI2, is widely disseminated via various plasmids [35, 36]. In our isolates, IncI2 was the most common (55.6%), followed by IncX4 (19.4%), IncHI2 (13.9%), and IncP1 (11.1%). IncI2 transfers DNA efficiently in the mouse gut [37]. Interestingly, it remains dominant in our study despite the absence of colistin use in any sampled population. This finding is consistent with reports that after colistin restriction measures, the proportion of IncI2 increases in *mcr-1*‐positive human isolates, suggesting its relative stability under nonselective pressure [27]. However, *mcr-1*‐harboring plasmids can be unstable in wild‐type *E. coli* and can be rapidly lost without colistin [38], implying a natural tendency toward disappearance. Thus, the persistence of IncI2 in egret‐associated isolates may facilitate the environmental transmission of *mcr-1* to crested ibises, while animal keepers could also act as carriers through environmental interactions. These findings highlight the importance of tracing resistance gene origins and transmission pathways in wild birds and reducing unnecessary interspecies interactions in captive habitats. Consistent with this, targeted interventions successfully eliminated *mcr-1* from the crested ibis population.

However, there are several limitations in this study. First, due to personnel constraints and the seasonal migration of egrets in the study region, we were unable to conduct seasonal sampling and systematic surveillance comparable to that performed for the crested ibis. As a result, our cross‐sectional survey was limited to the spring‐summer period, when egret activity was highest and personnel were available. Second, although a screening concentration of 4 mg/L colistin was applied, which aligns with commonly used selective protocols, this approach may not detect *mcr-1*‐positive isolates with lower MIC values [16]. Future studies using lower (e.g., 2 mg/L) or gradient concentrations of colistin could improve the detection sensitivity.

## 5. Conclusion

In conclusion, this study documents a temporally discrete outbreak of the plasmid‐mediated colistin resistance gene *mcr-1* in a captive population of endangered crested ibis. Integrated genomic and epidemiological evidence points to wild egrets as a critical vector for the environmental transmission of *mcr-1*‐bearing *E. coli*. The subsequent successful elimination of *mcr-1* from the crested ibis population following targeted biosecurity interventions underscores the effectiveness and practicality of such measures. Collectively, these findings highlight the importance of a one‐health approach that integrates wildlife surveillance into AMR containment strategies, particularly for safeguarding vulnerable species in shared ecosystems.

## Author Contributions

**Keyuan Chen**, **Yongchun Yang**, and **Wulin Ma**: conceptualization, writing − original draft. **Lei Lei**: methodology, investigation. **Guoqiang Qiu and Jian Shi:** data curation, formal analysis. **Ying Li and Yadong Zheng:** formal analysis. **Yongchun Yang, Houhui Song, and Weihuan Fang:** writing – review and editing, supervision. **Houhui Song**: funding acquisition.

## Funding

This work was supported by the National Key Research and Development Program of China (Grant 2023YFD1801005), the Natural Science Foundation of Zhejiang Province (Grant LQN25C180004), and the Zhejiang A&F University Talents Starting Program (Grant 2024LFR132).

## Ethics Statement

The whole operation procedures of the relevant experiments in this study were approved by the Ethics Committee of Zhejiang A&F University (ZAFUAC202464), and all of them have been carried out in accordance with the relevant provisions of the rules from the Chinese Center for Disease Control and Prevention.

## Conflicts of Interest

The authors declare no conflicts of interest.

## Supporting Information

Additional supporting information can be found online in the Supporting Information section.

## Supporting information


**Supporting Information 1** Table S1: Crested ibis fecal samples collected during 2017–2024.

**Supporting Information 2** Table S2: Resistance rates of *mcr-1*‐positive isolates tested by the Kirby‐Bauer disc diffusion method.

**Supporting Information 3** Table S3: Positive prevalence of *mcr-1* from 2021 to 2024.

**Supporting Information 4** Table S4: Isolation of *mcr-1* positive strains from the crested ibis breeding base in Deqing, China, 2021 (*n* = 144).

**Supporting Information 5** Figure S1: Genomic characteristics of the 36 *mcr-1*‐positive *Enterobacteriaceae* isolates. (A) Distribution of antibiotic resistance genes and (B) distribution of virulence genes. The molecular characteristics of each isolate are denoted by empty squares for absence and filled squares for presence.

**Supporting Information 6** Table S5: The *mcr-1* positive isolate information used in this study.

## Data Availability

The public accession number of sequence data is PRJNA1126054 in the manuscript.
