# Supplementary figures and images for: Egrets Promote the Transmission and Spread of Plasmid‐Mediated Colistin Resistance Gene mcr-1‐Bearing Escherichia coli Strains in Crested Ibis
Source: Transbound Emerg Dis. 2026 Jun 7;2026:4384955. doi: 10.1155/tbed/4384955 (PMC13243877; doi:10.1155/tbed/4384955)

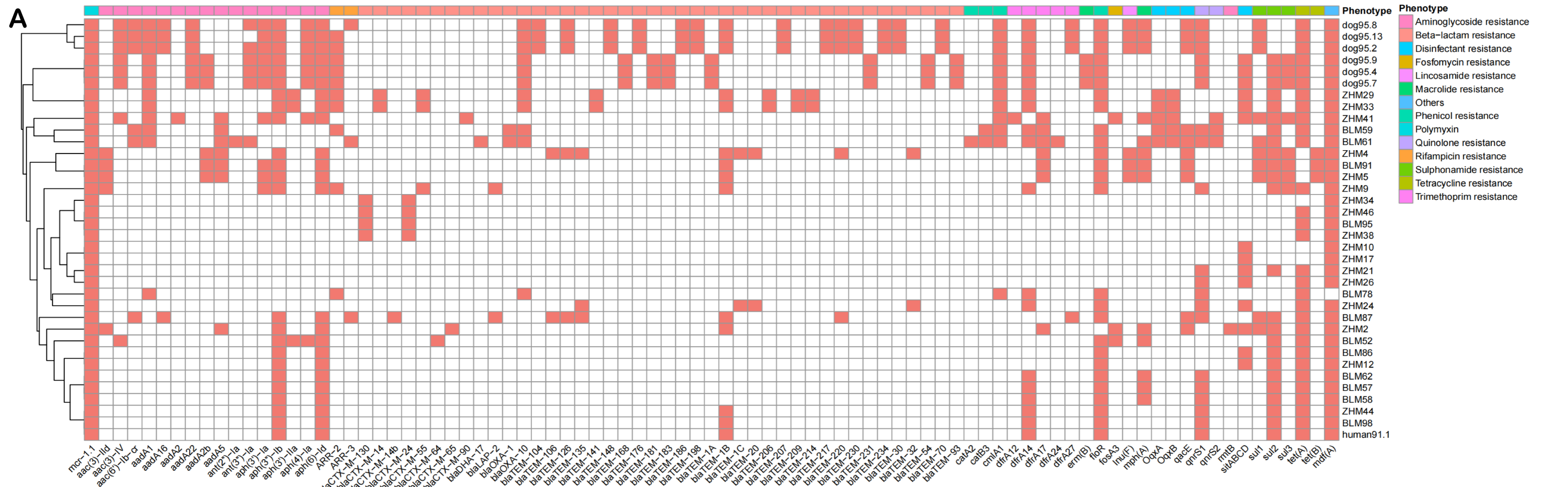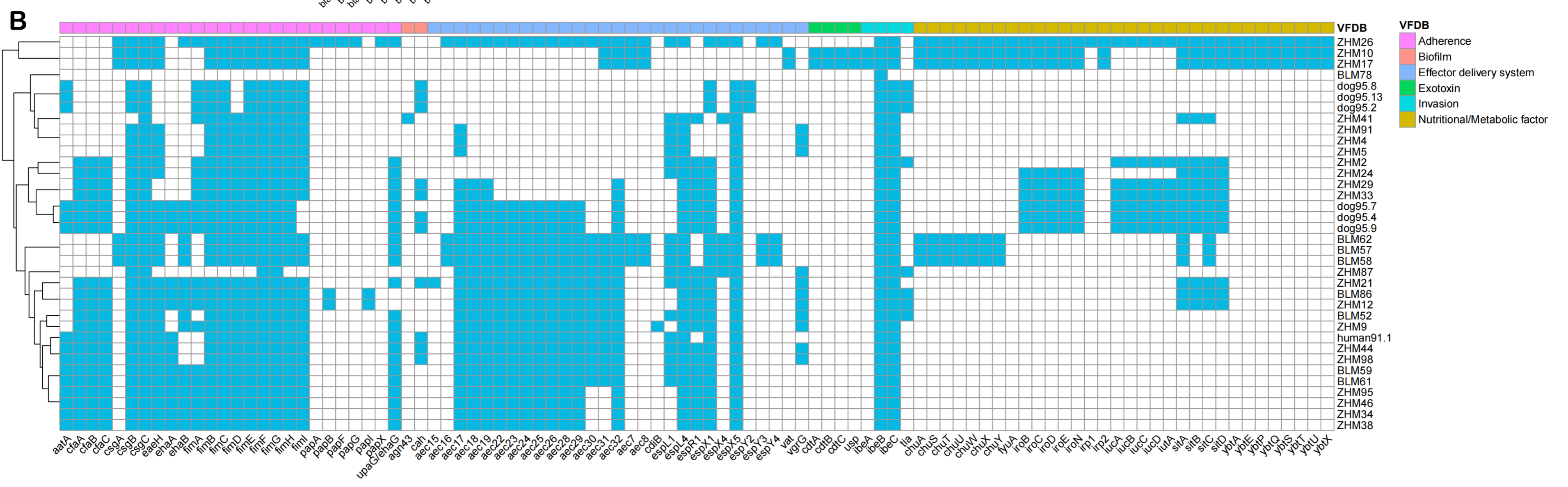

Supplement: Supplementary file 5 — Supporting Information 5 Figure S1: Genomic characteristics of the 36 mcr-1‐positive Enterobacteriaceae isolates. (A) Distribution of antibiotic resistance genes and (B) distribution of virulence genes. The molecular characteristics of each isolate are denoted by empty squares for absence and filled squares for presence. [file TBED-2026-4384955-s005.pdf]
